# Gait training with real-time augmented toe-ground clearance information decreases tripping risk in older adults and a person with chronic stroke

**DOI:** 10.3389/fnhum.2014.00243

**Published:** 2014-05-08

**Authors:** Rezaul K. Begg, Oren Tirosh, Catherine M. Said, W. A. Sparrow, Nili Steinberg, Pazit Levinger, Mary P. Galea

**Affiliations:** ^1^Gait and Balance Research Group, College of Sport and Exercise Science, Institute of Sport, Exercise and Active Living, Victoria UniversityMelbourne, VIC, Australia; ^2^Physiotherapy Department, Austin HealthMelbourne, VIC, Australia; ^3^Physiotherapy, The University of MelbourneMelbourne, VIC, Australia; ^4^Wingate College of Physical Education and Sport Sciences, Wingate InstituteNetanya, Israel; ^5^Department of Medicine (Royal Melbourne Hospital), The University of MelbourneParkville, Melbourne, VIC, Australia

**Keywords:** gait, augmented feedback, toe-clearance, tripping, stroke

## Abstract

Falls risk increases with ageing but is substantially higher in people with stroke. Tripping-related balance loss is the primary cause of falls, and Minimum Toe Clearance (MTC) during walking is closely linked to tripping risk. The aim of this study was to determine whether real-time augmented information of toe-ground clearance at MTC can increase toe clearance, and reduce tripping risk. Nine healthy older adults (76 ± 9 years) and one 71 year old female stroke patient participated. Vertical toe displacement was displayed in real-time such that participants could adjust their toe clearance during treadmill walking. Participants undertook a session of unconstrained walking (no-feedback baseline) and, in a subsequent Feedback condition, were asked to modify their swing phase trajectory to match a “target” increased MTC. Tripping probability (PT) pre- and post-training was calculated by modeling MTC distributions. Older adults showed significantly higher mean MTC for the post-training retention session (27.7 ± 3.79 mm) compared to the normal walking trial (14.1 ± 8.3 mm). The PT on a 1 cm obstacle for the older adults reduced from 1 in 578 strides to 1 in 105,988 strides. With gait training the stroke patient increased MTC and reduced variability (baseline 16 ± 12 mm, post-training 24 ± 8 mm) which reduced obstacle contact probability from 1 in 3 strides in baseline to 1 in 161 strides post-training. The findings confirm that concurrent visual feedback of a lower limb kinematic gait parameter is effective in changing foot trajectory control and reducing tripping probability in older adults. There is potential for further investigation of augmented feedback training across a range of gait-impaired populations, such as stroke.

## Introduction

The World Health Organization reports that falls injuries are the second leading cause of unintentional death after road accidents and are the major precursor to death in the elderly (WHO, 2002). Falls risk is considerably increased with ageing (Gillespie et al., 2003) but is substantially higher in stroke patients (AIHW, 2008; Batchelor et al., 2012). It is estimated that approximately 73% of stroke patients fall at least once in the first 6-months following discharge from inpatient rehabilitation (Forster and Young, 1995). The primary cause of falls-related injury is tripping (Cohen et al., 2003) due to unanticipated foot contact with ground-based objects, sufficient to irretrievably destabilize the individual (Nagano et al., 2011). Tripping is a hazard in the everyday environment either when walking over easily anticipated significant obstacles or lower but more frequently occurring surface irregularities, that may not always be accommodated by changes to swing limb trajectory. Goldie et al. (2000) found that in the homes of 22 stroke patients recently discharged from hospital, 2.5 to 4.4 obstacles were encountered for every 10 m of ambulation. Obstacles have been directly implicated in 10% of falls following stroke (Forster and Young, 1995). Foot trajectory during the swing phase of the gait cycle must not only maintain progression in the direction of travel, reflected in step length, but also incorporate a vertical displacement component sufficient to accommodate changes in support surface elevation.

**Figure 1:**
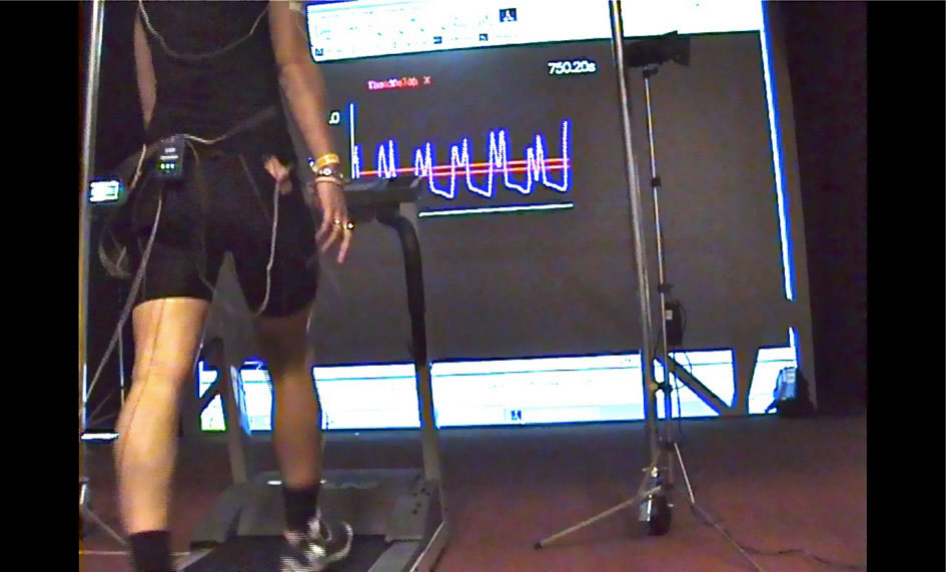
**Preferred-speed treadmill walking with visual augmented information of minimum toe clearance (MTC) provided using a real-time projection of the right foot sagittal trajectory**. The MTC target band established in a baseline trial is shown as two parallel lines and MTC is the low point between the two trajectory peaks.

Minimum Toe Clearance (MTC) is a critical event close to mid-swing in the walking gait cycle, when foot-ground clearance is minimal (1–2 cm). Low toe clearance at MTC has, therefore, been investigated as a predictor of tripping risk (Begg et al., 2007; Best and Begg, 2008). One gait adaptation to minimize tripping risk is to increase ground clearance at MTC while reducing variability. Recent work in our laboratory demonstrated that augmented vertical displacement information provided by projecting the real-time toe trajectory onto a screen (Figure 1), enabled young participants to both increase MTC and decrease variability (Tirosh et al., 2013). This gait-specific movement information (i.e., ground clearance at MTC) represents a general class of information provided by an external source, which supplements the performer's intrinsic, task-specific sensory information. The effectiveness of such “augmented information” for changing movement-related characteristics is well known in motor behavior research and the proposal that augmented information of kinematic variables could be used to optimize motor performance has also been long-established (Newell et al., 1983). A further consideration in designing the present experiment was that motor learning is operationally defined not only by the “relative permanence” of performance in a later retention condition (e.g., Salmoni et al., 1984; Sparrow and Summers, 1992) but also evidence of transfer of training from one action, to another similar but unpracticed movement. In this paper we measured gait cycle characteristics and toe height at MTC in both limbs, i.e., both the trained and untrained limb, to determine any evidence of transfer to the untrained foot's movement characteristics.

It is increasingly recognized in gait research that adaptive locomotor control is dependent on complex interactions between the lower limbs, as reflected in stride phase variables that are unequal or “asymmetrical” (Nagano et al., 2013). A further motivation for examining both limbs simultaneously was that with both ageing and gait pathology there is evidence of compensatory adaptations that protect the walker from balance loss, reflected in “functional” or adaptive changes to gait cycle variables. With ageing it has been suggested that the dominant limb adopts a primary role of forward progression while the non-dominant limb serves to secure gait stability (e.g., Sadeghi et al., 2000). It was of interest to identify changes to gait cycle parameters with training consistent with functional adaptations that would secure balance while also facilitating progression.

Visual augmented information has been demonstrated to improve balance and gait across a range of clinical populations such as children with cerebral palsy and people with multiple sclerosis (Basmajian, 1981; Seeger et al., 1981; Baram and Miller, 2006; Boonstra et al., 2008; Langhorne et al., 2011). Augmented feedback has also been adapted to gait therapy in adults with hemiplegia (Batavia et al., 2001). A review by Van Vliet and Wulf (2006) of extrinsic feedback and motor learning after stroke concluded that biofeedback improved longer term performance compared to no-feedback conditions. A recent randomized control experiment with stroke patients assessed the effectiveness of visual feedback for balance training (Rao et al., 2012). These researchers used foot-ground reaction forces from a force plate as biofeedback and showed larger gains in balance test scores and functional independence measures for the experimental group compared to no-feedback controls. This recent study provides evidence of accelerated positive effects of feedback training for gait retraining and the restoration of walking ability after stroke. Providing feedback of toe vertical displacement at MTC during walking may facilitate treadmill-based gait training in both healthy older individuals and older adults who have sustained neurological deficits due to stroke.

The research questions addressed here were first, whether, relative to an initial baseline condition, healthy older participants could increase toe height at MTC with augmented information using the real-time visual presentation procedure devised by our research group (e.g., Begg et al., 2012; Tirosh et al., 2013). The second question to be addressed was the feasibility of translating these procedures to neurological rehabilitation by applying similar augmented training procedures to an older individual who had sustained a stroke. Evidence of the feedback's effectiveness would be demonstrated first by improved performance in a post-training retention trial when augmented information was withdrawn and, second, evidence of greater vertical toe displacement at MTC in the untrained limb (i.e., transfer of training).

## Materials and methods

### Participants

Nine healthy community living older adults (aged 76 ± 9 years) and one 71 year old female who had sustained a stroke participated in the experiment. The stroke patient was 21 years post left sided hemorrhage, which resulted in right sided hemiplegia. She walked with a single point stick (gait speed 0.37 m/s). She had a Step Test score of 6 with the affected limb supporting and 0 with the unaffected limb supporting. She was unable to perform a Timed Chair Stand Test and had a Stroke Rehabilitation Assessment of Movement Lower Limb sub score of 13/20. She had no spasticity in gastrocnemius or soleus. All participants gave their written informed consent using procedures mandated and approved by the Victoria University Human Research Ethics Committee. In addition, the protocol for the stroke participant was approved by the Austin Hospital Human Research Ethics Committee.

### Data acquisition

All participants walked on a motor driven treadmill at preferred speed. Healthy older individuals performed three conditions within a single session; (i) 10 min walking (Baseline); (ii) 20 min walking with augmented MTC information (Feedback); (iii) 10 min walking with no feedback (Retention). The stroke patient completed training and testing over nine sessions, as follows: (i) 5 min walking (Baseline); (ii) eight sessions over 4 weeks with augmented MTC information (Feedback) which included four sessions of continuous feedback for 5 min and four sessions faded feedback (4 min feedback and 2 min no feedback); (iii) 5 min walking with no feedback (Retention), immediately post-training.

In Feedback the right toe trajectory was projected in real-time on a screen in front of the participants with instructions to elevate the right toe when walking (i.e., increase ground clearance at MTC) by maintaining vertical displacement within an upper and lower bound (bandwidth) superimposed on the trajectory. The right foot was the stroke patient's affected limb. The displayed target bounds were calculated from the Baseline trajectory data; the lower bound was 1.5 × mean toe height at MTC in Baseline and the upper bound was the lower bound mean + 3 × standard deviations of toe height at MTC in Baseline. In Retention all participants were asked to reproduce the Feedback toe height at MTC but no trajectory display was provided.

Three-dimensional lower limb position data were sampled at 100 Hz using two Optotrak Certus (Northern Digital Inc.) motion analysis camera units positioned on either side of the treadmill. Optotrak infrared emitting diodes (IREDS) were placed on the distal extremity of the 5th metatarsal head (toe) and the heel. Participants wore their own comfortable shoes to which heel and toe IRED markers were attached, representing the anatomical location. A static data sample was obtained to provide reference coordinates. All participants wore a safety harness.

### Data analysis

The Optotrak system provided x-y-z spatial coordinates of each marker with the x-axis parallel to the walking direction (anterior–posterior), the z-axis vertical, i.e., perpendicular to the treadmill belt and the y-axis lateral and perpendicular to the x and z axes (medio-lateral). The swing phase events right toe off (RTO) and right heel strike (RHS) were identified by applying gait event algorithms using the heel and toe velocity and acceleration (Zeni et al., 2008). Toe height at MTC was computed as the toe vertical local minimum between the first maximum following toe-off and the second maximum of vertical displacement (Nagano et al., 2011). MTC toe height descriptive statistics (mean, standard deviation, skewness, and kurtosis) for both feet were computed. Obstacle contact frequency was calculated as the probability of tripping (PT) over a 1 cm high obstacle using MTC distribution modeling devised by Best and Begg (2008) and then compared pre- and post-training to assess the effect of training on toe height at MTC.

To determine condition and foot effects on toe clearance at MTC for the older group the gait variables were entered into two One-Way repeated measures Multivariate Analysis of Variance (MANOVA) procedure, with condition (Baseline, Retention) and foot (Left, Right) the within-subject factors. ANOVA F-ratios with probability <0.05 were accepted as statistically significant. All statistical analyses were undertaken using *Statistica* (StatSoft Inc.).

## Results

Table 1 presents stride-cycle data for the older group and the stroke patient at baseline and in the retention trial following gait training with augmented toe-height information. ANOVA results indicated that for the older participants none of the walking cycle variables in Table 1 changed significantly from baseline to retention. In interpreting these results it is, however, worthwhile to note that these variables are influenced by walking velocity which was held constant across the two conditions (Older Group = 0.72 m/s; stroke patient = 0.28 m/s). Lower walking speed in the stroke patient was reflected in short steps of long duration relative to the older group. The stroke patient's hemiplegia was most clearly reflected in a right step (the affected limb) that was 10 cm longer than the left step. In addition, the stroke patient's right stance duration (as a percentage of stride time) was shorter (73%) than for the left foot (80%). The stroke patient's step width was 2–4 cm greater than the older group.

**Table 1:** **Gait cycle parameters for a group of older individuals and one stroke participant with an affected right limb**.

	**Older group**				**Stroke participant**			
	**Baseline**		**Retention**		**Baseline**		**Retention**	
	**Left foot**	**Right foot**	**Left foot**	**Right foot**	**Left foot**	**Right foot**	**Left foot**	**Right foot**
Stride length (m)	0.87	0.87	0.91	0.91	0.61	0.61	0.68	0.68
Stride duration (s)	1.21	1.21	1.25	1.25	2.21	2.19	2.46	2.44
Step length (m)	0.45	0.42	0.48	0.43	0.26	0.36	0.30	0.39
Step duration (s)	0.61	0.60	0.64	0.61	1.10	1.14	1.25	1.20
Step width (m)	0.21	0.21	0.20	0.20	0.23	0.23	0.23	0.24
Stance duration (%)	69	70	69	69	80	73	81	74

As illustrated in Table 2 the older group showed similar toe height at MTC for the two feet in baseline. Following training, as expected, higher mean right toe vertical displacement at MTC was seen in Retention (35.5 mm) compared to baseline (14.4 mm), with the ANOVA results confirming that the difference between these two means reflected a significant condition effect [*F*_(1, 32)_ = 6.96, *p* < 0.05]. For the stroke patient, toe-ground clearance in the affected right limb was 13 mm lower than for the left toe in Baseline, while in Retention the unaffected and non-treated left toe cleared the ground at MTC by 10 mm *more* than the feedback influenced right toe. This result confirmed that the trained foot toe height improved with feedback training. It is of further interest to note in Table 2 that the stroke patient's *unaffected* left toe height in Baseline was almost twice that of the older sample, while the affected right toe clearance (16 mm) was comparable to the older participants' toe-ground clearances.

**Table 2:** **Toe height characteristics at the Minimum Toe Clearance (MTC) swing-phase event**.

	**Older group**				**Stroke participant**			
	**Baseline**		**Retention**		**Baseline**		**Retention**	
	**Left foot**	**Right foot**	**Left foot**	**Right foot**	**Left foot**	**Right foot**	**Left foot**	**Right foot**
Toe height (mm)	15.3	14.4	19.5^*^	35.5^*^	29.1	16.6	34.9	24.9
Toe height *SD* (mm)	3.6	5.4	4.0	8.3	5.4	13.0	5.3	8.8
Skewness	1.20	−0.36	1.04	0.73	0.44	2.88	0.60	2.08
Kurtosis	1.66	1.38	1.58	1.60	0.29	11.27	−0.13	8.35
Contact frequency	2482	641	269	>10^5^	>10^5^	3	>10^5^	161

*SD is toe height standard deviation. Contact Frequency is the predicted frequency of toe contact with a 1 cm high obstruction at MTC, i.e., 641 represents predicted contact once in every 641 strides. Higher frequency represents lower tripping probability*.

* **p* < 0.05, Significant difference between Baseline vs. Retention conditions*.

While there was no condition effect on toe height variability in older participants, augmented information training of the right foot may have increased right toe-height variability relative to baseline. In contrast the stroke patient's right toe standard deviation *decreased* from 13.0 mm in Baseline to 8.8 mm in Retention. In addition to the toe height mean and standard deviation, the skewness and kurtosis characteristics of the toe-height distribution in Table 2 may have affected the predicted frequency of contact (for a 1 cm obstacle). The results show that all but one of the older subjects had positively skewed (*S* > 0; right skew) MTC and one other older person demonstrated negative kurtosis (*K* < 0) in the Baseline condition. Subject E3 had an exceptionally high negative skew (*S* = −8.9) to the right foot MTC that caused the overall group mean skew to be less than zero in Baseline. This subject's skew, however, became positive in Retention (*S* = 0.57). Subject E1 had negative Kurtosis (*K* = −4.4) which after training became positive (*K* = 3). Most interesting is the increase in predicted step frequency and associated reduction in probability of tripping (PT) from baseline to retention for all participants on the trained limb. The PT on a 1 cm high obstacle for the older adults reduced on average from 1 in every 578 strides to 1 in >10^5^ strides. With gait training the stroke patient's estimated tripping probability over a 1 cm obstacle at MTC was 1 in 3 strides at baseline and 1 in 161 strides immediately post-training.

**Figure 2:**
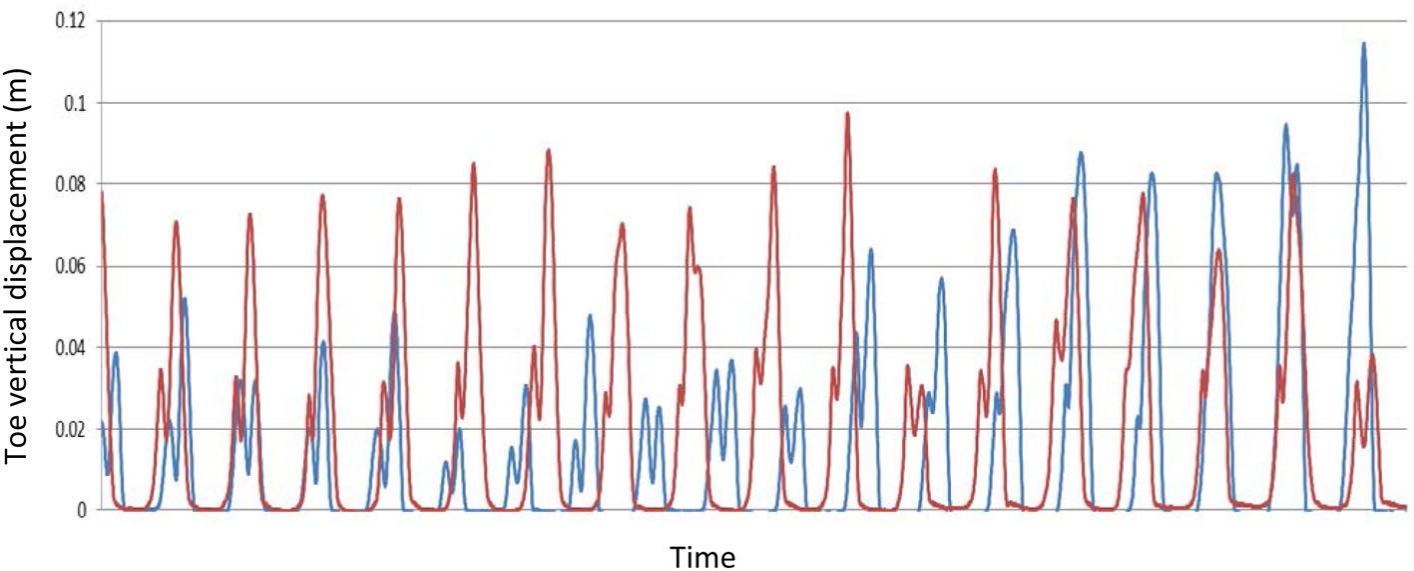
**A sample of the real-time vertical toe-ground displacement for the Stroke Participant during preferred speed treadmill gait training, Blue—Pre-training; Red—Post-training (Retention)**.

**Figure 3:**
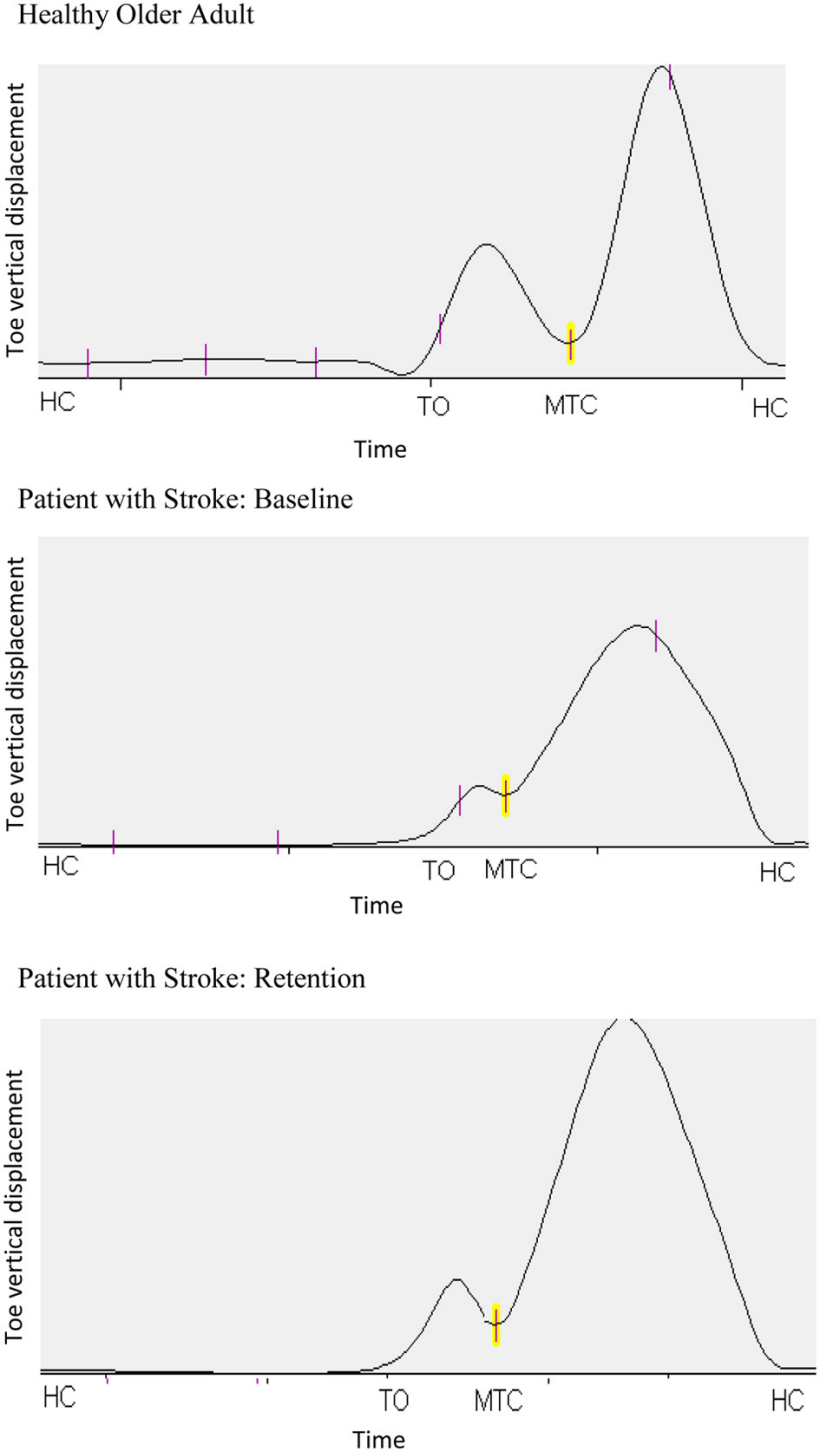
**Typical toe vertical displacement for one Healthy Older Adult and the Stroke Participant in pre-training Baseline and in Retention following training**. The plots are one complete time-normalized stride cycle, defined by consecutive heel contact (HC) events. The swing-phase is from the first HC to Toe-Off (TO) and MTC is the Minimum Toe Clearance event at approximately mid-swing.

Qualitative changes in the toe swing phase trajectory are illustrated in Figures 2, 3. Figure 2 shows the immediate effect of augmented information on the stroke patient's toe-trajectory control by comparing the Baseline and Retention toe-ground clearance time-series over multiple steps. It is noteworthy that MTC biofeedback tended to increase toe-ground clearance throughout the swing phase, not only at the targeted MTC event. Training effects on the stroke patient's lower limb trajectory control are highlighted further in the typical one gait cycle (Heel Contact to Heel Contact) plot in Figure 3. With training the plot more closely resembles that of the control older adult at the top of the figure with increased toe elevation and a more clearly defined MTC event.

## Discussion

Previous research has identified MTC at approximately mid-swing as a gait variable for predicting foot-contact with surface obstacles (e.g., Begg and Best, 2002). The aim of this experiment was to determine the efficacy of treadmill-based gait training with concurrent augmented information to increase toe height at MTC in older adults and reduce the predicted frequency of toe-ground contact. Consistent with the review by Barrett et al. (2010) Baseline MTC was in the range 10–20 mm for the older group and the stroke patient's *affected* limb MTC (16 mm) was comparable to the older adult data. Despite the stroke patient's significant gait impairment, as reflected in spatial and temporal gait cycle variables, it is interesting that high toe-clearance was maintained in both feet, perhaps a precautionary strategy but, toe-clearance was considerably greater in the unaffected limb.

When provided with augmented MTC information both the older group and the stroke patient maintained an elevated MTC in retention. Toe height variability in the stroke patient's affected limb reduced with feedback training while toe height at MTC increased. Increased toe height is associated with safer progression but in contrast to the stroke patient who decreased her right toe clearance variability from baseline to retention the older group increased variability in the right limb following training. As a consequence of increased Retention variability in the older group and variability *declining* with training in the stroke participant's treated right foot, toe-ground variability in retention was approximately equivalent. Positive skew indicates a distribution biased to the right (higher MTC), consistent with one of three strategies employed by the elderly to minimize tripping (Begg et al., 2007). The two other strategies to reduce tripping risk are either to increase MTC central tendency or reduce MTC variability (Begg et al., 2007; Best and Begg, 2008), the former observed in the present results following training.

Overall the above toe-ground clearance height results confirm that concurrent (“real-time”) visual information of lower limb kinematic gait parameters is effective in increasing anticipatory control of mid-swing toe height in both healthy older adults and the older adult with stroke. All participants reduced tripping probability in retention as reflected in an increased number of strides that would contact a 1 cm obstacle assuming no anticipatory corrections to toe-trajectory. The use of training using feedback to improve anticipatory (“feedforward”) control has been studied only to a limited degree (e.g., Tsao and Hodges, 2007). The mechanism underlying the improvement in MTC is yet to be elucidated, however it is possible that the augmented feedback of MTC plus task-specific training enhanced the internal model of gait to improve foot trajectory (Kawato, 1999). The results also revealed that the stroke patient's *un*affected left toe also increased height at MTC in Retention relative to baseline (29.1 and 34.9 mm, respectively). Changes to the kinematic characteristics of the untrained limb imply transfer of training, which could be very important in unilaterally affected populations. If transfer could be confirmed in future work with gait-impaired populations it would raise the possibility of training a patient's unaffected limb to induce positive effects on the injured or neurologically impaired contralateral limb.

The finding of longer stance duration (80% of stride time) in the stroke patient's non-affected limb (compared to 73% in the affected, right limb) and a spatially shorter left step suggest a stability-related adaptation, similar to that previously reported in older adults (Nagano et al., 2013). Step length of older adults' dominant limb was asymmetrically larger suggesting “functional asymmetry.” Older adults increased the proportion of step time in double support and reduced step length on the treadmill indicating adaptation that may preserve their balance (Nagano et al., 2013). Previous literature also reported asymmetry between the affected and non-affected limb's spatial-temporal parameters following stroke (e.g., Allen et al., 2011). Further evidence of the stroke patient employing gait adaptations to maintain stability is a greater step width (1–4 cm) than the older group.

Results are only available from one participant with stroke, which limits the generalizability of these results. However, the results are encouraging and support the feasibility of utilizing augmented feedback-based gait training to retrain walking following stroke. Future randomized controlled trials with appropriate sample size and power are required to demonstrate the effectiveness of this technique in people with stroke.

To complement biomechanical information concerning augmented information effects on the lower-limb's “end-point control,” reflected in toe height at MTC, it is also important to determine how hip, knee, and ankle joint angles are modulated to control MTC.

### Conflict of interest statement

The authors declare that the research was conducted in the absence of any commercial or financial relationships that could be construed as a potential conflict of interest.
